# Tet-Regulated Expression and Optical Clearing for In Vivo Visualization of Genetically Encoded Chimeric dCas9/Fluorescent Protein Probes

**DOI:** 10.3390/ma16030940

**Published:** 2023-01-19

**Authors:** Liliya Maloshenok, Gerel Abushinova, Natalia Kazachkina, Alexei Bogdanov, Victoria Zherdeva

**Affiliations:** 1 Bach Institute of Biochemistry, Research Center of Biotechnology of the Russian Academy of Sciences, 33, Bldg. 2 Leninsky Ave., 119071 Moscow, Russia; 2 Vavilov Institute of General Genetics of the Russian Academy of Sciences, 117971 Moscow, Russia; 3 Department of Radiology, UMass Chan Medical School, Worcester, MA 01665, USA

**Keywords:** dCas9 orthologs, doxycycline inducible Tet-On expression, fluorescent proteins, subcutaneous tumor xenograft, fluorescence imaging, FLIM, MRI, multimodal imaging, optical clearing, gadobutrol

## Abstract

The catalytically inactive mutant of Cas9 (dCas9) endonuclease has multiple biomedical applications, with the most useful being the activation/repression of transcription. dCas9 family members are also emerging as potential experimental tools for gene mapping at the level of individual live cells and intact tissue. We performed initial testing on a set of tools for Cas9-mediated visualization of nuclear compartments. We investigated doxycycline (Dox)-inducible (Tet-On) intracellular distribution of constructs encoding dCas9 orthologs from St. thermophilus (St) and N. meningitides (Nm) fused with EGFP and mCherry fluorescent proteins (FP) in human A549 cells. We also studied time-dependent expression of these chimeric fluorescent constructs (dCas9-FP) after Tet-On induction in live cells and compared it with the time course of dCas9-FP expression in experimental dCas9-FP-expressing tumor xenografts using a combination of fluorescence imaging and in vivo contrast-assisted magnetic resonance imaging for assessing the extent of tumor perfusion. In vivo Dox-induction of mCherry-chimera expression occurred in tumor xenografts as early as 24 h post-induction and was visualized by using optical clearing (OC) of the skin. OC via topical application of gadobutrol enabled high-contrast imaging of FP expression in tumor xenografts due to a 1.1–1.2-fold increase in FI in both the red and green channels.

## 1. Introduction

CRISPR/Cas9 was initially identified as a component of the adaptive immune defense system machinery of procaryotes [1,2]. It has subsequently been shown to be useful for introducing modifications to the eukaryotic genome in various model systems [3]. Eucaryotic genome editing via CRISPR/Cas9 relies on Cas9 endonuclease activity and the sufficiently high specificity of its targeting of DNA because of the dual sequence-specific mode of DNA recognition involving the protospacer-associated motif (PAM) and a 20-nucleotide single-guided (sg)RNA complementary to a genomic DNA target sequence [4]. The use of several Cas9 orthologs isolated from different bacterial sources differing in the use of PAM increases the number of possible sets of sequences for the specific assembly of dCas9. Furthermore, the spectrum of potential applications of Cas9 goes far beyond the generation of dsDNA strand breaks and their repair. Catalytically inactive (or nuclease-dead) dCas9 mutant specifically binds to dsDNA with no further cleavage [5]. Due to high DNA sequence specificity [6,7], dCas9 technology makes it possible to use multiplexing to target several genes simultaneously, affecting several genomic loci or several targets along one or more signaling systems involved in the specific pathology. For enabling easier, non-destructive detection of dCas9-targeted genomic DNA sequences, dCas9 can be fused with fluorescent proteins (FP) [8]. This line of work culminated in the development of the CRISPRainbow imaging tool, consisting of dCas9-FP as well as fluorescent sgRNAs [9]. A number of studies have involved the visualization of genomic loci in cells using dCas9 and included multiple optimization steps (e.g., sgRNA engineering and target validation [10]). The initial results in living cells indicated that three-dimensional diffusion of dCas9-FP had a key role during the “search” for mammalian genomic targets, including sub-second off-target binding events [11]. A breakthrough in spatiotemporal mapping with dCas9 in prokaryotes was achieved by applying sptPALM assays (an improved version of single molecule microscopy framework) and miCube [12]. The time scale of the target search and the intracellular concentration of Cas9 have been approximated; it takes 17 ± 4 ms per binding event between dCas9 and PAM, indicating the reversible binding of dCas9 to target sites that can still interfere with downstream bacterial plasmid replication [12]. The optimization of expression in mammalian cells could be potentially achieved by using dCas9 chimera expression under doxycycline-controlled Tet-Off or Tet-On lentiviral gene expression systems, which are commonly used in basic research as well as in biotechnology and gene therapy applications [13,14]. In vivo imaging of eukaryotic multicellular organisms is based on tomographic approaches [15,16] which potentially could be applicable for tracking genomic loci using dCas9-FP as an imaging tool, because high resolution microscopy on the whole-body level is not technically feasible. To perform visualization in situ or in vivo it is imperative to improve the detection and interpretation of specific optical signals generated due to the presence of the target sequences in the genome and/or their reciprocal orientation in the genome, which may be facilitated by using FRET (Förster resonance energy transfer). Well-separated donor and acceptor excitation spectra of the donor-acceptor pair, a long lifetime, a high photostability of the donor and a wide absorbance range of the acceptor favor efficient energy transfer that can be detected by change in fluorescence intensity [17,18]. Previously, FRET pairs of fluorescent proteins (FP) have been successfully used for detecting molecular events in mouse tumor xenografts [19,20]. Traditional planar imaging can help to estimate fluorescence intensity for quantitation of fluorophore concentration in the subcutaneous xenografts [21,22]. In this regard, EGFP/mCherry donor-acceptor FP pairs previously proposed for FRET experiments [17] could be used for imaging in vivo. A donor/acceptor FRET pair in the case of two or more dCas9 orthologues may assist in distance calculations [23]. However, so far, there have been few attempts to optimize chimeric fusion proteins for live cell imaging by combining different orthologs (e.g., NmdCas9 and StdCas9) for imaging genomic loci in living cells [9]. In contrast to in vitro systems, the detection of low-intensity specific fluorescence in living tissues is a complex task, but it may be assisted by increasing signal-to-noise ratios via optical clearing [24]. It has been demonstrated by us previously that some commercially available contrast agents, including Gadovist^TM^ (gadobutrol), provide optical clearing of skin and peripheral tissues [22,25]. Gadobutrol allowed the obtention of an optical image of increased contrast and its analysis using magnetic resonance imaging (MRI) of the same area of tissue or organ [26]. Therefore, the goals of our current research were: (1) to demonstrate the feasibility of Tet-On (doxycycline, Dox)-driven expression of dCas9-FPs in subcutaneous mouse tumor models; (2) to test the feasibility of a dCas9-FP pair of CRISPR/Cas orthologous chimeras for FRET imaging; (3) to improve specific imaging by using in vivo optical clearing agents and (4) to combine fluorescence intensity imaging (FI) with MRI-derived anatomical/tumor prefusion for providing in vivo mapping of the areas with Dox-driven chimera expression.

## 2. Materials and Methods

### 2.1. Construction of Cas9 Expression Plasmids

Human codon-optimized dCas9 (nuclease-dead) from *S. pyogenes* (Sp), *N. meningitidis* (Nm) and *S. thermophilus* (St) [27] were fused to 1×GFP and 1×mCherry, and then subcloned into FU-tet-o-hOct4 lentiviral transfer vector. The POU class 5 homeobox 1 (Oct4) in the FU-tet-o-hOct4 vector (Addgene plasmid No. 19778) was replaced by NmdCas9-mCherry (Addgene plasmid No. 64110), SpdCas9-EGFP (Addgene plasmid No. 64107), or StdCas9-EGFP (Addgene plasmid No. 64512), respectively, and the resulting constructs were designated as: Fu-tet-o-NmdCas9-mCherry, Fu-tet-o-SpdCas9-EGFP and Fu-tet-o-StdCas9-EGFP (Figure S1 in Supplementary Materials).

### 2.2. Production of Lentiviral Particles

FU-tet-o-StdCas9-EGFP, FU-tet-o-SpdCas9-EGFP and FU-tet-o-NmdCas9-mCherry were used to produce lentiviral particles by following a modified protocol [28]. The lentiviral particles were produced by co-transfection of 293 T cells with the highly purified lentiviral transfer plasmids, lentiviral envelope plasmid pCMV-VSV-G (Addgene plasmid No 8454) and lentiviral packaging plasmids pMDLg/pRRE (Addgene plasmid No 12251) and pRSV-Rev (Addgene plasmid No 12253). Transfections were carried out using Lipofectamine 2000 (Invitrogen), according to the manufacturer’s instructions. The cell culture supernatant containing viral particles was collected 48–72 h after the transfection, filtered through 0.45 mm polyvinylidene difluoride membrane filters (Millipore) and the sterilized solution was used to infect the cells.

### 2.3. Generating Stable Cell Lines with Tet-On Induction of Expression of Chimeras in Cells

A549 cells were obtained from the American Type Culture Collection (ATCC, Manassas, VG, USA) through Russian Cell Collection. Cells were grown in standard cell culture conditions at 5% CO_2_ and maintained in Dulbecco’s modified Eagle’s medium (Thermo Fisher Scientific, Waltham, MA, USA) supplemented with 10% fetal bovine serum (Bioclot GmbH, Aidenbach, Germany), 2 mM glutamine (PanEco) and penicillin/ streptomycin mix. A549 cells were transduced with resultant lentiviral particles. Dual expressing lines were obtained by co-transfection lentivirus with equal titer. After treatment on the 4th day with transduction by Dox (2 µg/mL) (Figure S2), cells were selected by single cell fluorescence sorting using a FACS Aria SORP cell sorter (Beckton Dickinson, Franklin Lakes, NJ, USA), plating a single cell in each well of a 96-well plate. We isolated single cells expressing dual chimeric (StdCas9-EGFP and NmdCas9-mCherry) proteins or s single chimera (StdCas9-EGFP or NmdCas9-mCherry only) (Figure S3), Clones were formed by expansion of single cells for about 14 days followed by establishing for 10 passages.

### 2.4. Fluorescence Microscopy and Time-Resolved Fluorescence Microscopy

Preliminary visualization of cells after inducing the expression of chimeras (StdCas9-EGFP and NmdCas9-mCherry) with Dox was performed using a ZOE™ Fluorescent Cell Imager (Bio-Rad, Hercules, CA, USA) equipped with LED (blue, green and red) illumination light sources with fluorescence detection in the green (excitation, 480/17 nm; emission: 517/23 nm) and red channel (excitation, 556/20 nm; emission, 615/61 nm). An inverted Zeiss Axio Observer Z1 microscope, equipped with a DI-AxioCam-HRm, was used for fluorescence cell imaging using an HBO 100 W/2 excitation source and appropriate filters (green cube Ex. BP 470/40, BS FT 495/Em. BP 525/50; red cube: Ex. BP 550/25 BS FT 570/Em. BP 605/70). Some images in vivo were acquired by using a Nikon Eclipse TE 2000 U (Nikon Instruments Inc, Melville, NY, USA) inverted microscope equipped with a DCS–120 Confocal Scanning System (Becker & Hickl GmbH, Berlin, Germany), which uses a multidimensional TCSPC technique described elsewhere [16]. A WL-SC-480-6 ps supercontinuum laser with an acousto-optic tunable filter AOTF-V1-D-FDS-SM (Fianium Ltd., Southampton, UK) was used to excite fluorescence. The emission of green fluorescence was selected by long pass HQ 500LP (Chroma Technology, Rockingham, VT, USA) and bandpass 525BP50 (Omega Optical, Bratellboro, VT, USA) filters and detected by an HPM-100-40 hybrid detector (Becker & Hickl GmbH, Berlin, Germany). Red fluorescence emission was selected by long pass HQ 550LP (Chroma Technology, Rockingham, VT, USA) and bandpass 620BP60 (Omega Optical, Bratellboro, VT, USA) filters and detected by an HPM-100-40 hybrid detector (Becker & Hickl GmbH, Berlin, Germany). For each time point, the data of three visual fields (3–20 cells) were processed at a constant fluorescence excitation time (10 s).

### 2.5. Subcutaneous Tumor Xenografts and Induction of Tet-O Expression of Chimeras In Vivo

All animal work was approved by the Bioethics Committee at the Research Center of Biotechnology RAS (Protocol N◦23/1, 16 June 2022) and was carried out according to the corresponding guidelines for animal use. Transduced human adenocarcinoma A549 (n = 6, male or female) and clone E9 (green-red chimera A549-StdCas9-EGFP-NmdCas9-mCherry) were used for tumor engrafting in mice. Tumors were obtained by inoculating subcutaneously 3 × 10^6^ cells per mice of 0.1 mL of DPBS Matrigel^TM^ in the right flank of animals. After subcutaneous injection of cells, we usually observed 1–2 nodules within several weeks. For optical imaging, the animals were anesthetized by using an intramuscular injection of 0.01 mL mixture of zoletil:rometar at 1:1 (*v*/*v*, Virbac Sante Animale, France) at 25 mg/mL and xylazine hydrochloride (20 mg/mL) 5–20 min prior to imaging. During MRI scanning, the animals were maintained at 37 °C under gas anesthesia (1.5–2% isoflurane in oxygen). An amount of 200 μg of doxycycline was administered by gavage in 200 μL of sterile water twice every 24 h. Tumor fluorescence was measured on the 1st, 3rd and 6th day.

### 2.6. Optical Imaging In Vivo

Measurements were performed in mice before doxycycline gavaging and on the 1st, 3rd and 6th days after Dox induction. Whole body images were acquired by the imaging system iBox (UVP, Upland, CA, USA) equipped with a 16-bit monochrome CCD (BioChemi HR Camera, UVP, Upland, CA, USA) and a 150-W halogen quartz lamp (BioLite, UVP Upland, CA, USA). The in vivo fluorescence imaging was obtained using band-pass excitation (502–547 nm) and an ethidium bromide emission filter (570–640 nm) for mCherry chimera and band-pass excitation (440–470 nm) and a GFP emission filter (510–560 nm) for EGFP chimera. The image acquisition time was 1–10 s. FLIM of mouse tumor xenografts were acquired by using a Nikon Eclipse TE 2000 U inverted microscope (Nikon Instruments Inc., Melville, NY, USA) equipped with a DCS–120 Confocal Scanning System (Becker & Hickl GmbH, Berlin, Germany) setup, based on a multidimensional TCSPC technique described earlier [29]. A WL-SC-480-6 ps supercontinuum laser with an acousto-optic tunable filter AOTF-V1-D-FDS-SM (Fianium Ltd., Southampton, UK) was used to excite the fluorescence. The emission of green fluorescence was selected by the long pass HQ 500LP (Chroma Technology, Rockingham, VT, USA) and bandpass 525BP50 (Omega Optical, Bratellboro, VT, USA) filters and detected by an HPM-100-40 hybrid detector (Becker & Hickl GmbH, Berlin, Germany). The emission of red fluorescence was selected by the long pass HQ 550LP (Chroma Technology) and bandpass 620BP60 (Omega Optical) filters and detected by an HPM-100-40 hybrid detector (Becker & Hickl GmbH, Berlin, Germany).

### 2.7. Optical Clearing with Gadobutrol

Gadobutrol (1 M) (Gadavist^®^, Bayer HealthCare Pharmaceuticals, Berlin, Germany) was diluted to form a 0.7 M gadobutrol solution with addition of 5% dimethyl sulfoxide (DMSO) and 25% water (ACS, analysis grade), according to [22]. Obtained optical clearing composition (OC) was equilibrated for 1 h at room temperature and then used for skin topical application. OC mixtures were applied for 15 min onto the skin surface above the tumors. After topically application of OC, compositions were carefully absorbed using a cotton swab and the images were recorded. Optical contrasting images were registered after 30 min of gadobutrol application.

### 2.8. Magnetic Resonance Imaging

MRI (1 T ^1^H) (M3™ Compact High-Performance MRI System, Shoham, Israel) with a 50 × 150 mm inner bore and equipped with 440 mT/m gradients was used for mouse MRI. The mice were imaged by using a body RF coil (diameter 38 mm, length 50 mm) with an integrated heating system. Pre- and post-OC topical application images was obtained by using T1-weighted 3D gradient-echo (GRE) sequences (FOV 40 × 40 mm, 256 × 256 imaging matrix at TR/TE = 60/2.9 ms, FA 20°, NEX 5 (12 min scanning time)) after selecting 1 mm-thick coronal slices (n = 6–14). Maximum intensity projection (MIP) images were generated by using MRI slice stacks (n = 4–5). MIP images were used for registration with FI images (Image Fiji, National Institutes of Health, Bethesda, Rockville, MD, USA).

### 2.9. Statistical Analyses

FI was measured in 2–3 ROI per tumor (Figure S4). by using image segmentation (ImageJ/Fiji) with thresholding [30]. The differences in fluorescence intensity and values before and after OC were considered statistically significant (*p* < 0.05) according to a one-tailed non-parametric Mann–Whitney–Wilcoxon (MWW) *t*-test paired observation and data were analyzed using a one-tailed non-parametric (unpaired) MWW *t*-test, with n = 6–12 data points per group. Multiple groups were compared using ordinary one-way ANOVA test and the ANOVA Kruskal–Wallis test (Prism 9, GraphPad, San Diego, CA, USA).

## 3. Results

### 3.1. dCasCas9-FP Chimera Expression in Cell Culture

**Figure 1:**
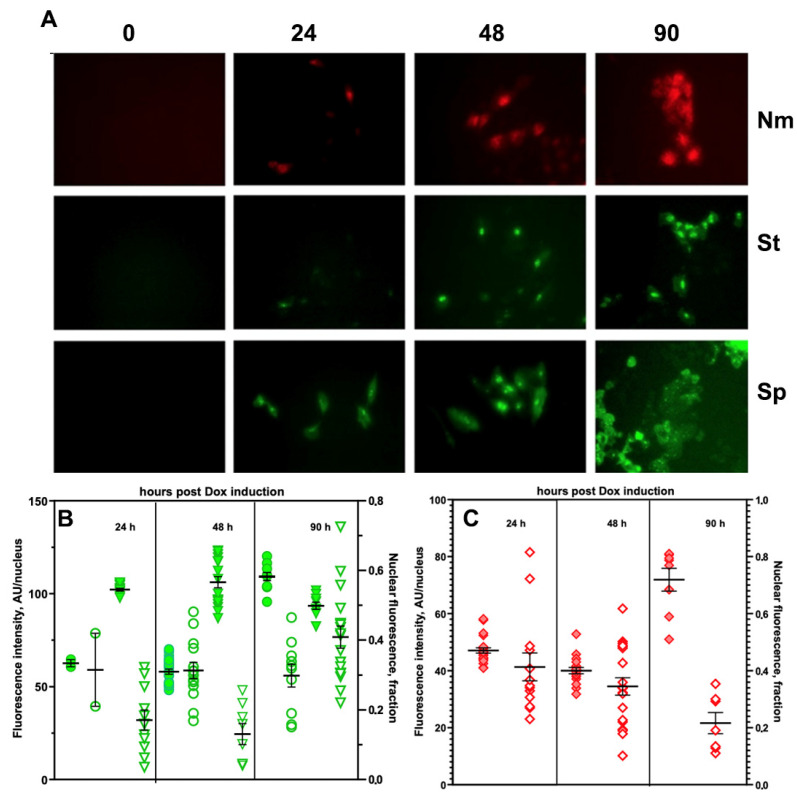
Tet-On-induced expression of chimeras by doxycycline (2 μg/mL) at 0 h, 24 h, 48 h and 90 h post induction. (**A**) Fluorescence microscopy of dCas9-FP chimeras expressed in A549 cells: (1) NmdCas9-mCherry (top row); (2) StdCas9-EGFP (middle row) and (3) SpdCas9-EGFP (bottom row) after Dox-induced expression. EGFP fluorescence: Ex. BP 470/40, BS FT 495/ Em. BP 525/50; mCherry fluorescence: Ex. BP550/25 BS FT 570/Em. BP 605/70 (Zeiss Axio Observer, X40). (**B**) Green FI dependence in cell nuclei due to expression of StdCas9-EGFP (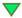) or SpdCas9-EGFP (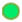) A549 cells, as well asthe fraction of total fluorescence in cell nuclei after the induction (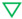, 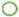); (**C**) the dependence of red FI of cell nuclei (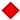) due to expression of NmdCas9-mCherryand the fraction of total FI of the cell nuclei (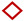) after theinduction of chimera expression by Dox.

FP expression in LV-transduced cell culture was observed on the day after Dox induction. The fluorescent signal progressively decreased during the 4–5 d following the withdrawal of Dox (Figure 1). Fluorescent microscopy of the A549 cells revealed the presence of fluorescent products after 24 h in the presence of Dox, which confirms the induction of chimera expression in cells expressing recombinant rtTA activator. The latter was introduced into the cells by using a lentiviral construct carrying a puromycin resistance marker for cell selection. According to Figure 1A, the development of fluorescence intensity (FI) was observed for all three dCas9 orthologs, with both FI and the number of positive cells increasing over time (from 24 to 90 h). Cell microscopy allowed for quantitative image processing to determine FI by image segmentation. An analysis of both the average nuclear FI and the nucleus/cell ratio was necessary to optimize the conditions for further experiments with the visualization of induced expression, which can be performed not only under cell culture conditions, but also in vivo. The calculation of total and average values of FI showed that there were clear differences between StCas9-EGFP and SpdCas9-EGFP, both in the kinetics of fluorescence changes and in the total level of FI. In the case of StCas9-EGFP, a steady increase in the accumulation of the fluorescent chimera in the nuclei was observed over time, while the nucleus/cell FI ratio remained approximately the same (Figure 1A,B, green circles). In the case of SpdCas9-EGFP, we observed a more rapid increase in FI 24 h post-induction, with decreased transport to the nucleus over time and with the accumulation of the fluorescent product in the cytoplasm (Figure 1A,B, green triangles), which resulted in a drop in the nucleus/cell FI ratio. In the case of chimeras of red FP (mCherry), the measurements of FI and its intracellular distribution resembled the expression of StCas9-EGFP. It should be noted that 90 h post-Dox induction, there was a strong 2–fold increase in FI with a parallel increase in the number of organelles containing the cytoplasmic form of NmdCas9-mCherry (Figure 1A,C, red diamonds).

**Figure 2:**
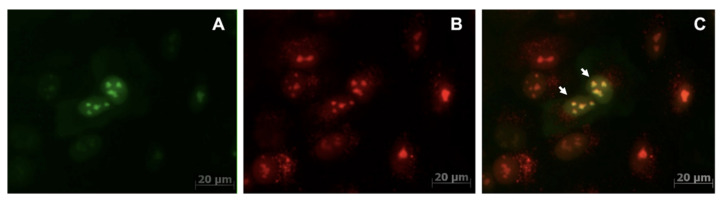
Fluorescence microscopy of A549 cells expressing Tet-On-driven St dCas9-EGFP/Nm dCas9-mCherry chimeras after Dox-induced expression: (**A**) Nm dCas9-mCherry (red channel Ex. BP 550/25 BS FT 570/Em. BP 605/70); (**B**) StdCas9-EGFP (green channel Ex. BP 470/40, BS FT 495/ Em. BP 525/50) and (**C**) StdCas9-EGFP/NmdCas9-mCherry (dual channel Ex. BP 470/40, BS FT 495 Em. BP 605/70) (Zeiss Axio Observer, X40). Arrows point to cell nuclei with dual FP-chimera expression.

**Figure 3:**
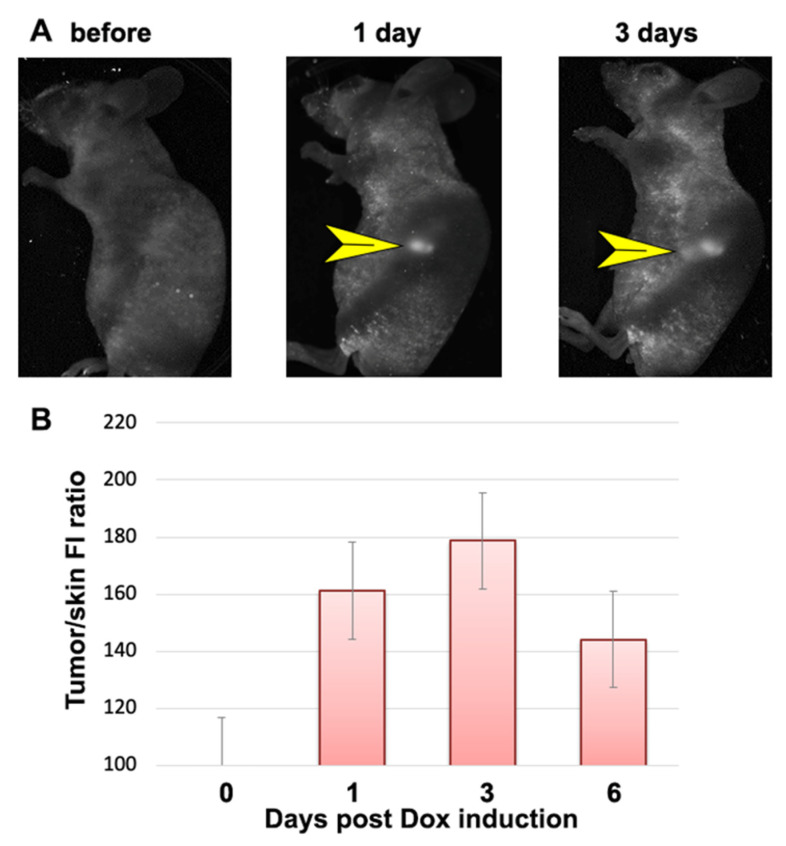
Tet-On Dox-induced fluorescence of dual dCas9 chimeras expressed in E9-derived tumor xenograft (A549-StdCas9-EGFP-Nm/dCas9-mCherry) after two doses of 200 μg of Dox/animal: (**A**) In vivo FI images after the induction of Nm dCas9-mCherry chimera expression before Dox induction (day 0) and on the 1st and 3rd day after Dox induction. Yellow arrowheads point to the fluorescent tumor; (**B**) Normalized red FI measured in E9 tumor xenograft after the induction of expression of chimeras before induction (day 0) and 1–6 d post-Dox-induction. Excitation: 502–547 nm, emission: 570–640 nm.

Thus, quantitative measurements of nuclear FI changes after transduction and the morphology of the distribution of fluorescence showed that: (1) the optimal time (time window) for monitoring the co-expression and co-localization of fluorescent chimeras of dCas9 was 24–48 h and (2) StdCas9-EGFP chimera can be used as a preferred donor of excitation photons in a combination with NmdCas9-mCherry as a FRET pair donor. Cells expressing StdCas9-EGFP (Figure 2A) and NmdCas9-mCherry (Figure 2B) showed normal morphology with predominantly nuclear dual fluorescence in co-transfected cells after LV transduction. Dual expression of both chimeras was present in about 20% of all cells (Figure 2C). Sorting of single cells enabled obtaining a set of clones (e.g., E9 used for in vivo work, Figure 3) with subsequent stabilization in cell culture after 10 passages.

### 3.2. In Vivo Detection of dCas9-FP Expression

To test the feasibility of inducible dCas9 chimera expression imaging in experimental tumors, xenografts were propagated in athymic mice in vivo after subcutaneous injection of A549 (clone E9) cells. Cells showed Dox-dependent expression of dual chimeras (StdCas9-EGFP and NmdCas9-mCherry) after the animals were given doxycycline (200 μg 2 × daily) on the second week post-implantation. The initial induction of expression was followed by FI measurements with 3 d intervals in between the measurements, i.e., on the 1st day (24 h), 3rd day (72 h) and on the 6th day (148 h) post-Dox-induction (Figure 3A). Figure 3A shows the mean FI of the tumor normalized by the reference FI value of the skin. On the first day, the tumor/skin (signal/background ratio, expressed in % of ratio increase) was affected by the background FI of the skin (Figure 3B). However, no “leaky” expression of FP chimeras in the absence of Dox was observed. Dox-induced expression of FP chimeras in tumor xenografts was observed 24 h post-induction [31]. The corresponding increase in tumor FI was 1.6-times on the 1st day, 1.8-times on the 3rd day, with a subsequent decrease in 1.4 × times the initial FI recorded on the 6th day after Dox induction (Figure 3B). The FI increase over time in cell culture differed from the in vivo kinetics. This can be explained by the uneven distribution of Dox in the tumors in vivo and partial metabolism and excretion in contrast to Dox-induction in the cell culture. To improve the quality of planar imaging, which had insufficient resolution and contrast, we used OC by applying a 0.7 M gadobutrol (GB) solution onto the skin as previously described [22].

**Figure 4:**
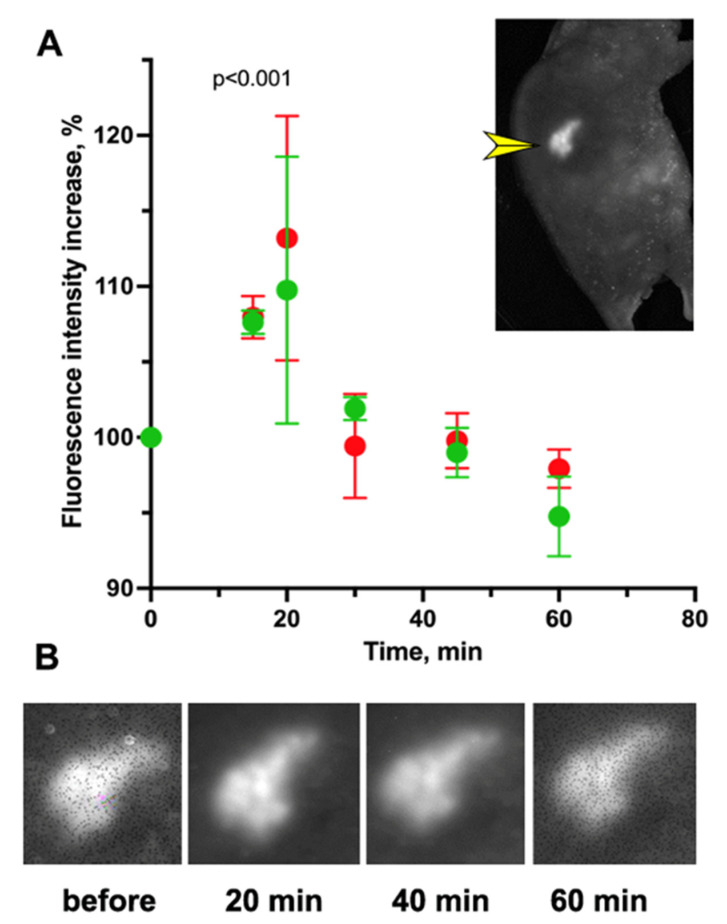
Gadobutrol (GB)-assisted OC effect in E9 tumor xenograft (A549-St dCas9 EGFP-Nm/ dCas9-mCherry) after Dox-induced chimera expression in mice on the 3rd day; (**A**) E9 tumor/to skin normalized mCherry fluorescence on the 3rd day in two animals (red and green) after Dox induction and using 0.7 M GB as an OC agent before and after (15–60 min) GB application, *p* < 0.001 (Wilcoxon–Mann–Whitney test, n = 6–12/time points, data shown as mean ± SD). Inset: mouse planar fluorescence imaging (ex 502–547 nm, em 570–640 nm) at the optimized time point chosen from FI dependence on time. Yellow arrowhead points to a subcutaneous tumor; (**B**) Tumor FI images which correspond to the time points on the graph (**A**) reflecting the kinetics of FI changes.

The improvement in tumor optical image quality after GB application is shown in Figure 4 (compared with Figure 3A). Post-induction tumoral chimera expression based on the FI showed an increase of 10–14%, as determined after GB application (Figure 4B). Figure 4B shows that the maximum increase in FI was observed at 15–30 min after the application of GB. The measured FI value showed a decrease to the initial level after 45–60 min, with visible contrast improvement in the tumor image (Figure 4B), which is consistent with previously reported data [22].

**Figure 5:**
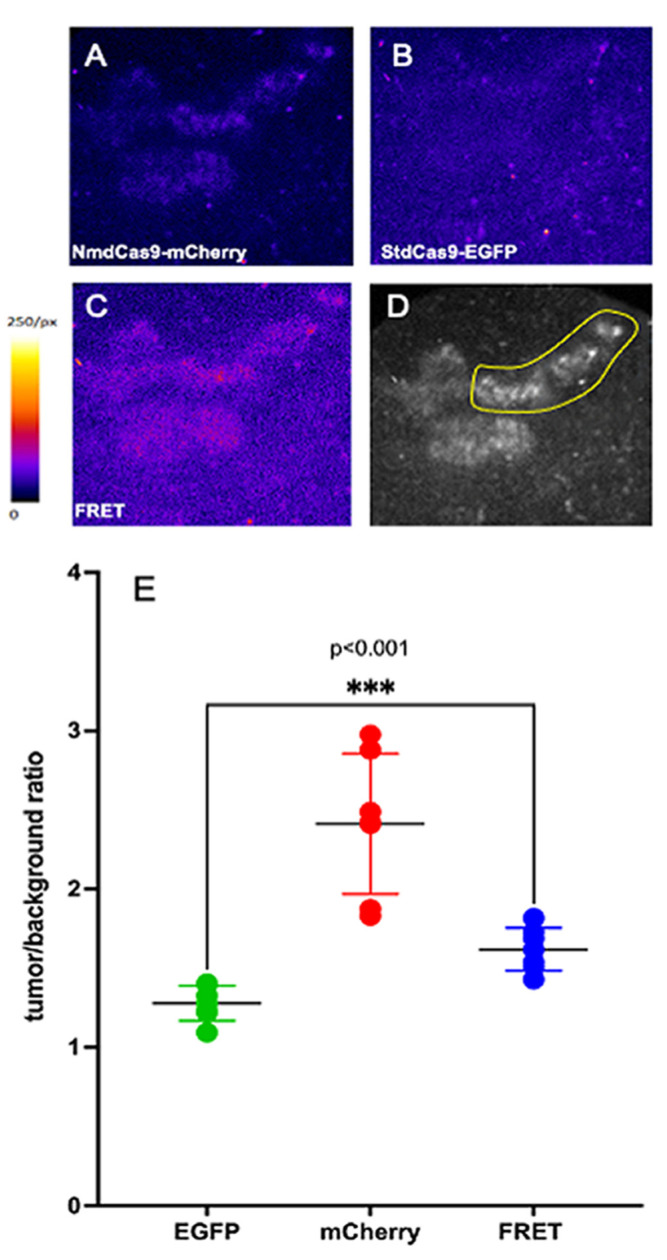
Fluorescence of subcutaneous E9 tumor xenograft (A549 StdCas9-EGFP-NmdCas9-mCherry) on the 3rd day after Dox induction (2 × 200 μg via gavage) and at 15 min application of 0% GB solution. FI images were obtained using a confocal scanner. Excitation wavelengths: 480 nm (donor) and 540 nm (acceptor). (**A**) Fluorescence intensity imaging of NmdCas9-mCherry (AOFT) 540 nm, HQ 550LP (Chroma) and 620BP60 nm (Omega); (**B**) FI imaging of StdCas9-EGFP (AOFT) 480 nm, HQ 500LP (Chroma) and 525BP50 (Omega); (**C**) FI imaging of FRET (AOFT) 480 nm, HQ 500LP, 620BP60 nm) donor: StdCas9-EGFP and acceptor: NmdCas9-mCherry. In (**A**,**B**), the FI is shown in a pseudocolor scale (Image J/Fiji); (**D**) FI intensity image showing ROI selection for quantitation; (**E**) Tumor-to-skin FI ratio measured for single orthologs (StdCas9-EGFP) (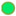) and NmdCas9-mCherry (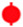) as well as in FRET-mode (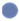). Data are shown as mean ± SD (n = 2–3 measurements in n = 3 animals), *p* < 0.001(Wilcoxon–Mann–Whitney test, n = 6 data points).

The use of a confocal imaging system allowed macro-imaging of tumors with spectral resolution of donor, acceptor and FRET. FI of chimeras are shown in Figure 5A–D. Figure 5E shows the tumor/background FI ratio for each channel measured over several tumor-specific ROI. Both donor FI and FRET FI ratios were significantly higher than the FI of the donor (*p* < 0.001).

**Figure 6:**
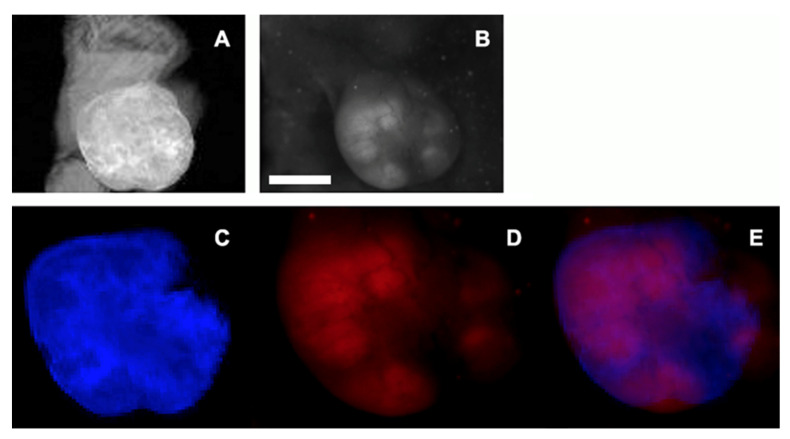
Representative MRI and FI imaging results obtained in an E9 xenografted mouse tumor. (**A**) 3D GRE MR image (1 T (Aspect Imaging), TR/TE = 60/2.9 ms, FA 20°, NEX = 5) 30 min after the application of 0.7 M GB, maximum intensity projection image obtained using a stack of four MRI slices; (**B**) planar FI imaging of NmdCas9-mCherry ortholog chimera; (**C**–**E**) a pseudo color image showing the overlay (**E**) of MRI (**C**, blue) and FI (**D**, red) of E9 clone-based tumor xenograft in the animal injected with 0.15 mmol/kg GB I.V.

The use of total tumor volume imaging by using contrast-assisted MRI enabled the detection of tumor perfusion with an MR contrast agent (gadobutrol) (see representative image, Figure 6A). By fusing a gadobutrol-enhanced MRI maximum-intensity pixel projection image of tumoral perfusion with a tumor FI obtained in the red FI emission channel (i.e., NmdCas9-mCherry expression in tumor tissue), we performed mapping of the tumor peripheral volume that had both perfused areas and areas of chimera expression. As expected, GB perfusion-delineated tumor regions coincided with Dox-induced dCas9-FP chimera expression areas (Figure 6).

## 4. Discussion

The main goal of this work was to test fluorescent imaging as a potential tool for inducible expression of catalytically inactive dCas9-FP in cell cultures and experimental animals, and to study intracellular redistribution of dCas9-FP after induction. The novelty of our research is in the development of using of FRET for detecting co-localized and/or genomic DNA-interacting dCas9-FP chimeras via conditional gene expression in living cells.

**Figure 7:**
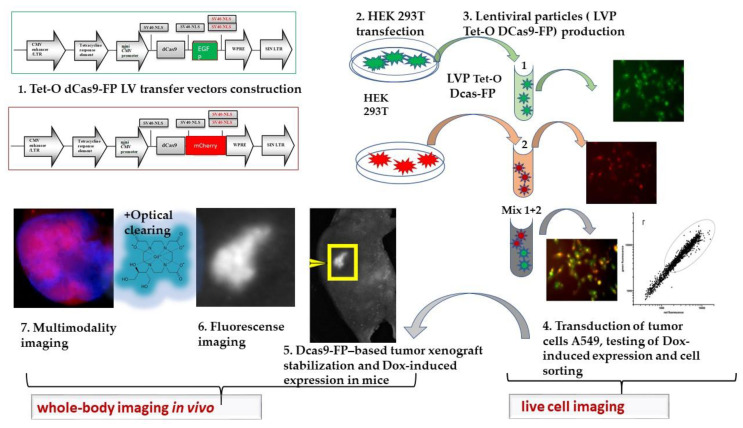
Optimized protocol for dCas9-FP chimera generating, monitoring of Tet-On expression in vitro and in vivo followed by FI and FRET measurements and MRI.

The protocol for modified dCas9-FP chimera engineering and monitoring of Tet-On expression in vitro and in vivo followed by FI and FRET measurements and MRI has been optimized as shown in the schematic chart (Figure 7).

We chose dCas9 methodology based on previously reported applications for multi-color DNA loci imaging in vitro (Ma et al. [9]). We tested Dox-inducible expression of three available dCas9 orthologs (i.e., previously described chimeras of SpdCas9, St1dCas9 (1109 AA) and NmdCas9 (1122 AA)) optimized for LV-mediated expression, which involved cloning of inserts that were almost 1 kb shorter than traditional SpdCas9 cDNA. In contrast to the “classical” NGG PAM DNA sequence, which is recognized by SpCas9, StCas9 recognizes the NNAGAAW sequence, while NmCas9 specifically binds to the NNNNGATT sequence as PAM [31,32,33,34]. Given the possibility of using PAM sequences in both (+) and (−) orientations on complementary DNA strands, the choice of at least three dCas9 available orthologs and large numbers of brightly fluorescent FP facilitates creation of a variety of FRET pair combinations with various mutual orientations and linear distance from each other along the genomic DNA sequence. To select conditions for further experiments both in vitro and in vivo, we followed the FI of FP, which starts to develop in the cells and tissues due to Dox induction made possible by engineering of the rtTA binding sequence in the regulatory region of the minimal promoter to enable Tet-On expression. The absence of cell fluorescence before induction with Dox proved the tight inducible regulation of dCas9-FP chimera expression. Our results showed similar FI distribution for StdCas9 and NmdCas9 orthologs of the NLS-fused dCas9 chimeras [35] in the absence of cellular sgRNA expression (Figure 1). The use of multiple copies of fluorescent proteins sequentially fused together with dCas9 [9] was found unnecessary in the case of FRET, especially in the case of green fluorescent proteins with high quantum yield [17]. In addition, despite significant gains in signal intensity, triple chimeras with fluorescent proteins mature more slowly in the cell and may undergo misfolding; therefore, they may be toxic [36,37]. Preliminary imaging of inducible ortholog expression in the cells demonstrated separate ortholog localization as well as it’s presence at the overlapping sites in the nuclei.

Optical imaging of FI provides high spatial resolution, high speed and specific molecular contrast; however, at the same time, it poses a limited probing depth and field of view and suffers from light scattering [18,24,38]. However, low-intensity fluorescence signals could be amplified with the aid of optical clearing (OC) of the skin [24,25]. As demonstrated by us previously, the FI of red chimera (dCas9-mCherry) in an LV-transduced E9 clone (A549 StdCas9-mCherry/ NmdCas9 EGFP) was amplified two-fold in vivo by applying 0.7 M gadobutrol (Gadovist^TM^), used as a clinical MRI contrast agent for OC due to its high refractive index [22]. Similar effects were observed in alternative subcutaneous tumors expressing red fluorescent TagRFP protein [22,26]. The observed dual effect of GB as an OC agent (Figure 5) and as an MRI contrast agent for tumor perfusion imaging (Figure 6) provided initial evidence that is important for future multi-modality imaging experiments. A single contrast agent for MR imaging improved both the intensity and contrast of FI after local application, while systemic injection of GB was shown to aid in mapping of tumor perfusion by MRI.

## 5. Conclusions

The feasibility of engineering stable cell lines expressing inducible dual (red and green fluorescent) chimeric dCas9-based proteins has been demonstrated. Tet-On transcriptional activation of fluorescent chimera expression by doxycycline administration allowed the regulation of the level of fluorescence over time in vivo. We described novel aspects of the regulated dual expression of dCas9-FP chimeras under the control of Tet-On regulatory elements and the possibility of choosing the time-window for their combined visualization in cells as well as in tumor xenografts. Dox inducible expression prevented the accumulation of the expressed products in the cytoplasm over time. The use of the optical clearing approach enabled the high-contrast imaging of FP expression in tumor xenografts. The dual effect of GB as an OC agent and as an MR contrast agent shows promise for further research involving fluorescence and MRI bimodal imaging, since the same MRI contrast agent improved FI and contrast of optical imaging and, in addition, enabled tissue perfusion imaging by MRI. Down the line, a combination of sgRNA expression with inducible dCas9-FP expression will provide the necessary toolbox for highly specific FRET-based imaging approaches, allowing the visualization and mapping of specific markers within genomic loci.

## Data Availability

Not applicable.

## Supplementary Materials

The following supporting information can be downloaded at:https://www.mdpi.com/article/10.3390/ma16030940/s1, Figures S1–S4: title Figure S1: FU-tet-o-SpdCas9-FP vector plasmids; Figure S2: Fluorescence microscopy of A549 cells expressing Tet-On-driven St1dCas9-EGFP/Nm dCas9-mCherry chimeras after Dox-induced expression; Figure S3: Single-cell sorting of A549 cells expressing Tet-On-driven St1 dCas9-EGFP/Nm dCas9-mCherry chimeras after Dox-induced expression; Figure S4: Fluorescence intensity imaging of NmdCas9-mCherry and 620BP60 nm with manual ROI in subcutaneous E9 tumor xenograft (A549 StdCas9-EGFP-NmdCas9-mCherry) on the 3rd day after Dox induction.
