# Supplementary material for: Tet-Regulated Expression and Optical Clearing for In Vivo Visualization of Genetically Encoded Chimeric dCas9/Fluorescent Protein Probes
Source: Materials (Basel). 2023 Jan 19;16(3):940. doi: 10.3390/ma16030940 (PMC9918104; doi:10.3390/ma16030940)
Supplement: Supplementary file 1 [file materials-16-00940-s001.zip › materials-2010747-supplementary.pdf]

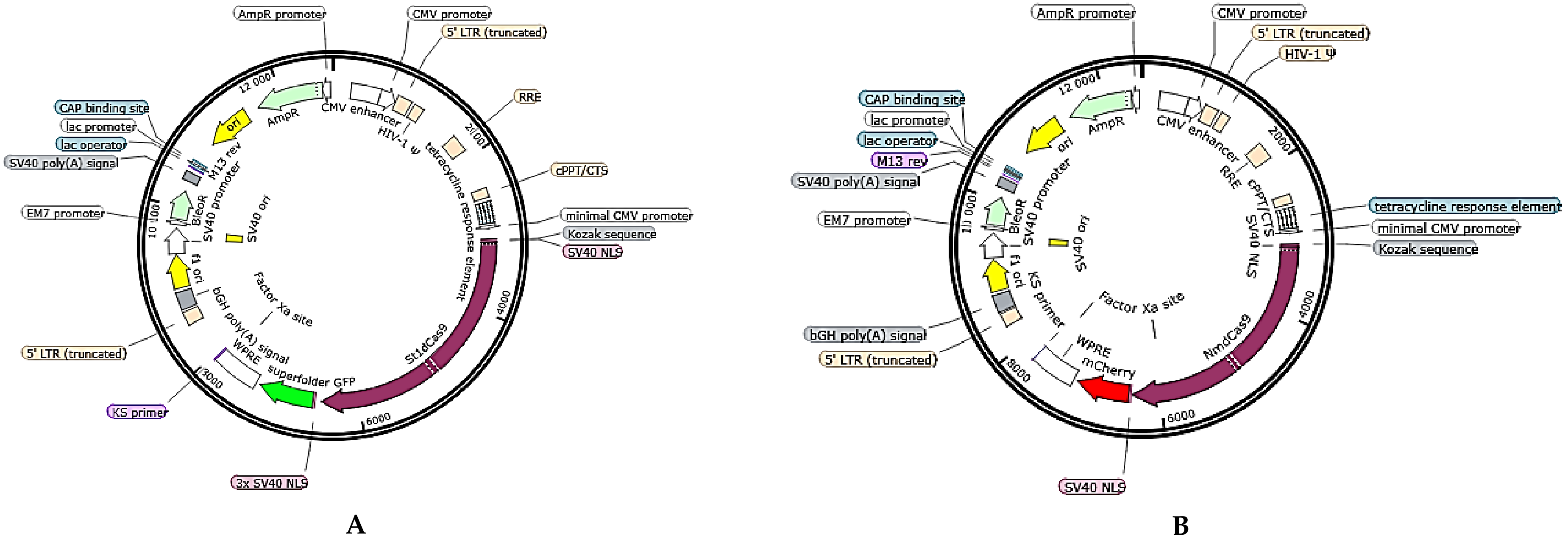

Figure S1. FU-tet-o-SpdCas9-FP vector plasmids.

(A) FU-tet-o-St1dCas9-EGFP vector plasmid; (**B)** FU-tet-o-NmdCas9-mCherry vector plasmid.

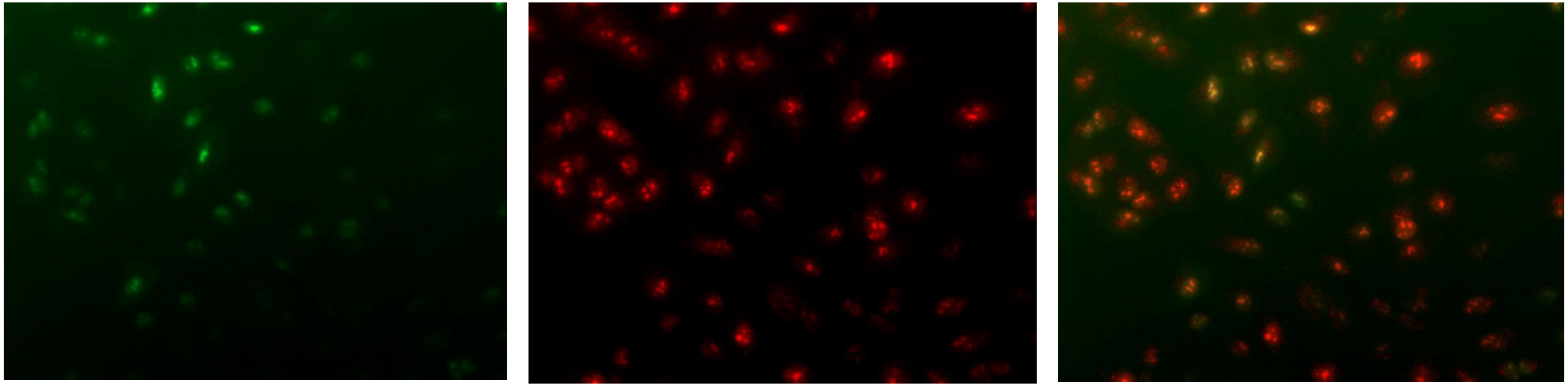

A B C

Figure S2. Fluorescence microscopy of A549 cells expressing Tet-On-driven St1 dCas9-EGFP/Nm dCas9-mCherry chimeras after Dox-induced expression: **(A)** Nm dCas9-mCherry (red channel Ex. BP 550/25 BS FT 570/Em. BP 605/70); **(B)** St1dCas9-EGFP (green channel Ex. BP 470/40, BS FT 495/ Em. BP 525/50) and **(C)** St1dCas9-EGFP/NmdCas9-mCherry (dual channel Ex. BP 470/40, BS FT 495 Em. BP 605/70) (Zeiss Axio Observer, X40).

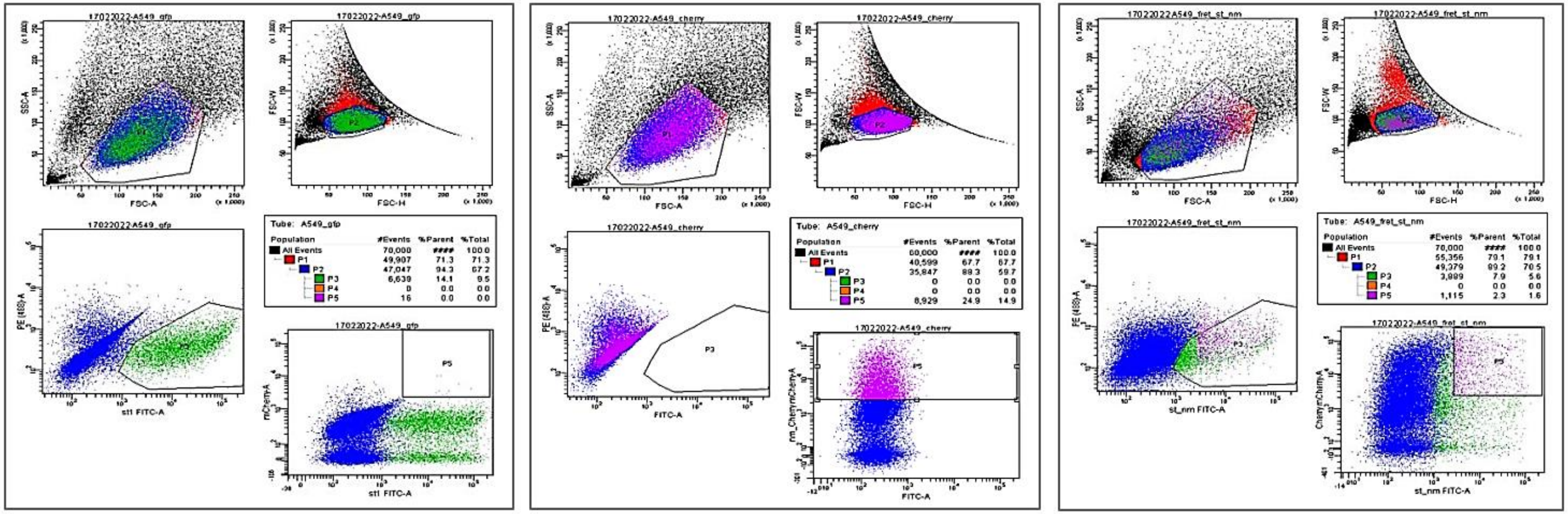

A B C

Figure S3. Single-cell sorting of A549 cells expressing Tet-On-driven St1 dCas9-EGFP/Nm dCas9-mCherry chimeras after Dox-induced expression: (**A)** St1dCas9-EGFP (green channel); (**B**) Nm dCas9-mCherry (red channel); (**C)** St1dCas9-EGFP/NmdCas9-mCherry (dual channel). FACS Aria Sorter (Beckton Dickinson).

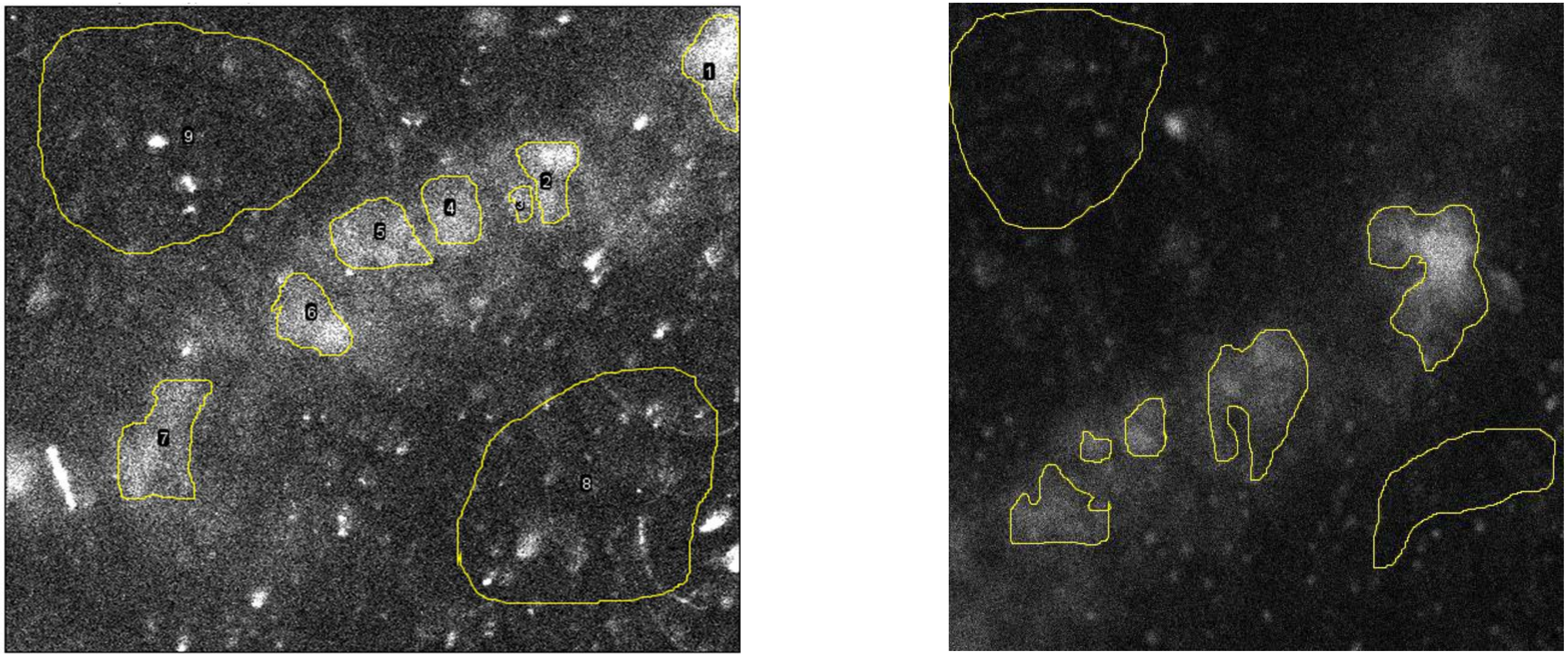

A B

Figure S4. Fluorescence intensity imaging of NmdCas9-mCherry (AOFT 540 nm, HQ 550LP (Chroma) and 620BP60 nm (Omega) with manual ROI in subcutaneous E9 tumor xenograft (A549 StdCas9-EGFP- NmdCas9-mCherry) on the 3rd day after Dox induction (2 × 200 μg via gavage) (**A)** before and (**B)** after 15 min application of 70% GB solution.
